# DP-YOLO: A Lightweight Real-Time Detection Algorithm for Rail Fastener Defects

**DOI:** 10.3390/s25072139

**Published:** 2025-03-28

**Authors:** Lihua Chen, Qi Sun, Ziyang Han, Fengwen Zhai

**Affiliations:** 1 School of Information Science & Technology, Southwest Jiaotong University, Chengdu 611756, China; clh@crscd.com.cn; 2 School of Electronics and Information Engineering, Lanzhou Jiaotong University, Lanzhou 730070, China; hanziyang20220222@163.com (Z.H.); zhaifw@mail.lzjtu.cn (F.Z.); 3 CRSC Research & Design Institute Group Co., Ltd., Beijing 100070, China

**Keywords:** rail fastener defects detection, lightweight, YOLOv5s, attention mechanism, statistical information weighted feature maps, 68T07

## Abstract

To enable accurate and efficient real-time detection of rail fastener defects under resource-constrained environments, we propose DP-YOLO, an advanced lightweight algorithm based on YOLOv5s with four key optimizations. First, we design a Depthwise Separable Convolution Stage Partial (DSP) module that integrates depthwise separable convolution with a CSP residual connection strategy, reducing model parameters while enhancing recognition accuracy. Second, we introduce a Position-Sensitive Channel Attention (PSCA) mechanism, which calculates spatial statistics (mean and standard deviation) across height and width dimensions for each channel feature map. These statistics are multiplied across corresponding dimensions to generate channel-specific weights, enabling dynamic feature recalibration. Third, the Neck network adopts a GhostC3 structure, which reduces redundancy through linear operations, further minimizing computational costs. Fourth, to improve multi-scale adaptability, we replace the standard loss function with Alpha-IoU, enhancing model robustness. Experiments on the augmented Roboflow Universe Fastener-defect-detection Dataset demonstrate DP-YOLO’s effectiveness: it achieves 87.1% detection accuracy, surpassing the original YOLOv5s by 1.3% in mAP0.5 and 2.1% in mAP0.5:0.95. Additionally, the optimized architecture reduces parameters by 1.3% and computational load by 15.19%. These results validate DP-YOLO’s practical value for resource-efficient, high-precision defect detection in railway maintenance systems.

## 1. Introduction

Railway fasteners, as a crucial component of the track structure, directly impact the safety and stability of railway operations [1]. In recent years, with the rapid development of computer vision and deep learning technologies, automated defect detection methods based on image processing have gradually become a research hotspot in the field of Structural Health Monitoring (SHM) [2]. As a key part of SHM, the technological advancement of railway fastener defect detection is of great significance for enhancing the overall effectiveness of the monitoring system. With the maturation of deep neural networks and the emergence of large-scale public datasets for track fastener defect detection, deep learning-based algorithms for track fastener defect detection have demonstrated better robustness and higher detection accuracy compared to traditional methods, and are thus often better able to meet the needs of practical applications.

The problem of track fastener defect detection falls under the category of specific object detection tasks. Deep learning-based object detection algorithms are divided into two main categories: two-stage and one-stage. Two-stage object detection algorithms primarily consist of two steps: region proposal generation and classification regression. One-stage object detection merges the region proposal and object classification or regression steps into a single pass through the neural network.

The development of Convolutional Neural Network (CNN) architectures has gone through several stages of improvement, including Region-based Convolutional Neural Network (RCNN) [3], Fast R-CNN [4], Faster R-CNN [5], Mask R-CNN [6], and Cascade R-CNN [7], and so on, which are all classic two-stage models.

One-stage object detection algorithms use regression to predict detection boxes directly, rather than generating candidate regions, thereby simplifying the detection problem into a regression task [8]. One-stage object detection algorithms include YOLO [9], YOLO9000 [10], SSD [11], YOLOv3 [12], YOLOv4 [13], and YOLOv5 [14], among others.

Both two-stage and one-stage object detection algorithms have made significant progress in various detection tasks, including the problem of track fastener defect detection. Wei Feng [15] and others proposed a lightweight object detection model called YOLOv5_SS, which uses the Soft-NMS algorithm to improve the detection of densely overlapping objects. Li H et al. [16] enhanced the extraction of key features by improving the attention mechanism. Zou et al. [17] treated pixels outside the region of interest as negative samples, and by introducing an additional loss function, they were able to suppress the extraction of irrelevant features by the Backbone, thereby enhancing the effective feature representation.

Although object detection algorithms have achieved some research results in track fastener defect detection, there are still some problems at present, such as false detection, missed detection, and repeated detection of multi-scale and occluded targets. In addition, track fastener defect detection algorithms also need to meet real-time requirements while ensuring detection accuracy.

The two-stage R-CNN series methods, such as R-CNN, Fast R-CNN, and Faster R-CNN [18], and Faster R-CNN, face difficulties in real-time applications for track fastener defect detection due to their insufficient real-time performance. In contrast, the one-stage algorithms, such as the YOLO series, which have been continuously improved, demonstrate good real-time performance and have shown excellent performance in object detection. However, there are still issues in practical deployment, such as large model size, numerous parameters, unsatisfactory real-time performance, and reduced robustness in complex scenes. YOLOv5s, YOLOv8s, and YOLOv10s are three commonly used lightweight object detection models. They have achieved a good balance between speed and accuracy, making them suitable for scenarios that require rapid detection. The performance comparison of YOLOv5s, YOLOv8s, and YOLOv10s (YOLOv11s has not been released yet) is shown in Table 1.

As can be seen from Table 1 that YOLOv8s and YOLOv10s have achieved further improvements in terms of accuracy and speed compared to YOLOv5s. However, due to yolov5s’s low computational complexity (FLOPs, low energy consumption), good stability, ease of deployment, mature community, and rich documentation support, YOLOv5s remains a research focus for many object detection studies. Furthermore, the backbone network and feature fusion network of YOLOv8 and YOLOv10 are optimizations based on YOLOv5. Concurrently, many existing defect detection methods are based on the idea of combining object segmentation with object recognition. However, bounding box detection methods offer several notable advantages, including real-time performance and efficiency, feasibility for subsequent quantitative processing, and simplicity and efficiency in data annotation and processing [19,20]. Therefore, based on the YOLOv5s model, this paper proposes a lightweight track fastener defect detection algorithm: DP-YOLO (DSP + PSCA YOLO). The main contributions of this paper include the following four aspects:

**Table 1:** Performance Comparison of YOLOv5s, YOLOv8s, and YOLOv10s.

Feature	YOLOv5s	YOLOv8s	YOLOv10s
Release Time	2020	2023	2024
Parameters	7.2 M	11.2 M	7.2 M
FLOPs	17.0 G	28.6 G	21.6 G
COCO mAP	37.2%	44.9%	Higher (specific values to be released)
Inference Speed	Fast	Faster	Fastest (optimized computational efficiency)
Use Cases	Embedded devices, mobile, real-time detection	High-precision real-time detection, large-scale video analysis	High-precision real-time detection, large-scale video analysis
The complexity of deployment	It supports exporting models in multiple formats, which enables it to be easily deployed across various hardware platforms, including mobile devices, edge devices, and servers.	Although it also supports exporting in multiple formats, more optimization and adaptation work may be required during the actual deployment process.	Although it has significant performance improvements, its new architecture and training methods may require more optimization and adaptation work to achieve optimal performance across different platforms.
Community Support and Documentation	It enjoys extensive community support and abundant documentation resources, which enable developers to quickly find solutions and resources to address problems.	Although its community is also growing, compared to YOLOv5s, its community resources and documentation may not be as rich.	As a newer version, its community support and documentation resources are still being continuously improved.

First, the DSP (Depthwise Separable convolution Stage Partial) module was designed. This module uses Depthwise Separable Convolution (DSC) [21] and conventional convolution to build the W3_D module, which replaces the Bottleneck in the C3 module of the original YOLOv5s. This improves the detection capability for multi-scale and occluded objects while significantly reducing computational costs and the number of model parameters.

Second, the Position Sensitive Channel Attention (PSCA) module was designed. This module generates weights for each channel feature map based on the mean and standard deviation of the horizontal and vertical dimensions of each channel and then performs a weighting operation on the feature map. This enhances the model’s ability to perceive important features. By inserting the PSCA module before the spatial pyramid pooling layer in the backbone network, the model’s feature representation capability is improved.

Third, the Neck part was designed with lightweight architecture, integrating the C3Ghost module, which effectively reduces network parameters and computational load. At the same time, the Alpha-IoU loss function is introduced to accurately measure the overlap between predicted boxes and ground-truth boxes, thereby further improving the model’s detection accuracy for objects of different scales.

Fourth, the experimental dataset originates from the Fastener-defect-detection Dataset on Roboflow Universe [22], which comprises 2234 images. Given the small size of the dataset and the imbalance in data categories, this paper employs a random combination of various enhancement methods to diversely augment and expand the dataset. Subsequently, annotations were made for the expanded 6702 images, ultimately increasing the experimental dataset size to 8936, with the training set comprising 6520 images and the test set consisting of 2416 images.

The original dataset (2234 images) was split into a training set (2061 images) and a test set (173 images). To address data scarcity and class imbalance, augmentation techniques. Each training image was augmented using three randomly selected methods, expanding the training set to 6520 images and the training set to 2416. The augmentation methods include adding noise, changing brightness, cropping, translating, rotating, mirroring, and cutout. In order to increase the diversity of the samples, each image was augmented using three randomly selected methods from the aforementioned techniques. Subsequently, extensive ablation and comparative experiments were conducted on the augmented dataset, which validated the effectiveness of the proposed method in this paper.

## 2. The Basic Framework of YOLOv5

**Figure 1:**
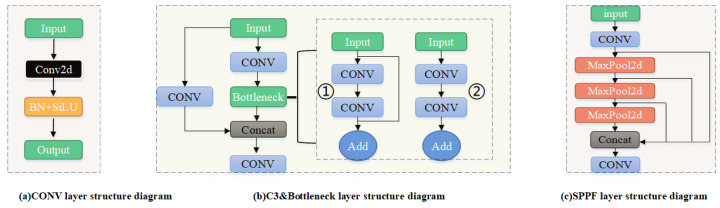
Schematic diagram of each module of YOLOv5s backbone.

YOLOv5 consists of a Backbone, a Neck for feature fusion, and a Head for prediction. It employs an improved CSP Darknet53 structure. The Backbone is mainly composed of CONV layers, C3 layers, and a Spatial Pyramid Pooling Fusion (SPPF) layer. The CONV layer consists of three components: the Conv2d convolution function, the BN normalization function, and the SiLU activation function, as shown in Figure 1a.

The C3 layer is an improved version of the CSPBottleneck layer [23]. It contains three convolutional layers and several Bottleneck modules, as shown in Figure 1b. There are two versions of the Bottleneck module. One has a residual structure, as shown in Figure 1b➀, and the other does not have a residual structure, as shown in Figure 1b➁. This paper mainly adopts the Bottleneck with the residual structure.

The SPPF (Spatial Pyramid Pooling Fusion) layer is located after the last C3 layer in the backbone. It extracts contextual information at different scales through a factorized spatial pyramid pooling approach, thereby enhancing the model’s receptive field. This leads to more accurate and stable object detection, as shown in Figure 1c.

The feature enhancement part is composed of PANet [24], which can improve the network’s ability to fuse features and obtain richer target information. The prediction part of the Head layer in YOLOv5 mainly includes three prediction heads, each of which generates feature maps of three different sizes.

## 3. DP-YOLO Network Module

**Figure 2:**
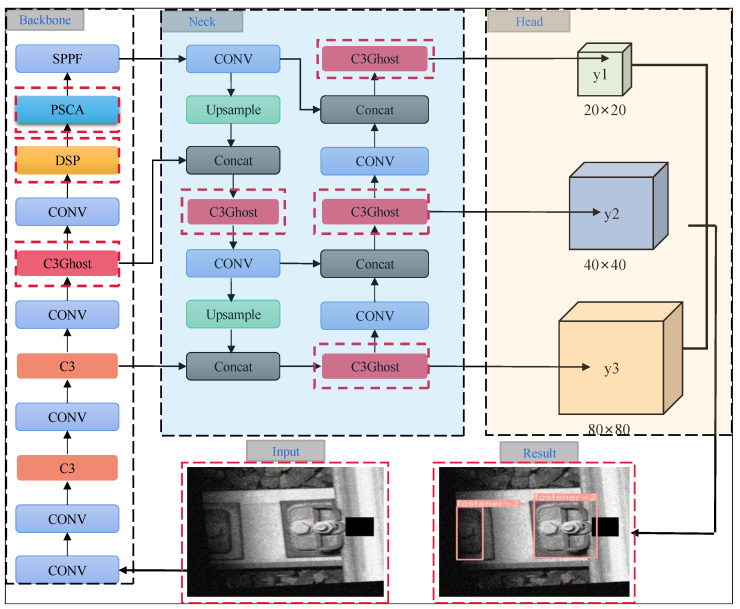
The DP-YOLO Network Structure.

The network model structure of the proposed DP-YOLO in this paper is shown in Figure 2. The input image size of the network is 640 × 640. After processing, three feature maps of different scales, namely y1, y2, and y3, are obtained, with sizes of 20 × 20, 40 × 40, and 80 × 80, respectively. By utilizing the multi-scale feature maps output by the network, the algorithm is capable of handling fastener targets of small, medium, and large scales. This enhances the efficiency of target recognition across different scales and further improves the accuracy of detection. The backbone network of this paper employs the DSP module (DSC stage partial) and the Position Sensitive Channel Attention mechanism to ensure that the backbone network can better extract key features. The red dashed boxes indicate the main differences between this paper and the YOLOv5s module, with detailed explanations provided in the later subsections. The following sections introduce the main modules of the proposed model.

### 3.1. Design of the DSP Module Based on Depthwise Separable Convolution

The C3 module refers to the CSPC3 (CSP Bottleneck with 3 convolutions) module, which is an important component in the YOLO model. Its function is to extract semantic features at multiple scales. The C3 module consists of one Bottleneck and three convolutions. Each Bottleneck contains two convolutional layers. The Bottleneck employs a CSP (Cross Stage Partial) structure, which fuses information from feature maps of different levels to enhance the expressiveness and discriminability of the features. Moreover, the CSP structure can significantly reduce the computational load of the model, thereby enhancing its trainability and generalizability.

**Figure 3:**
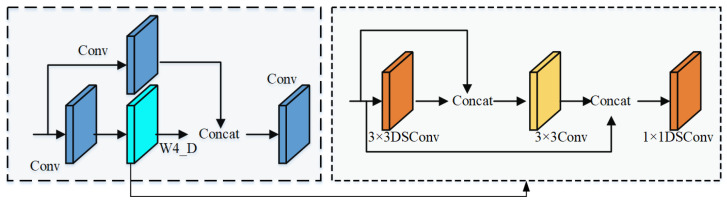
DSP&W3_D Structure Diagram.

Despite the many advantages of the C3 module, its accuracy in detecting railway fasteners still needs to be improved. Therefore, this paper enhances its feature representation capability by improving the Bottleneck in the C3 module. The DSP (DSC stage partial) module designed in this paper is shown in Figure 3, where a newly constructed W3_D module is used to improve the Bottleneck in the C3 module.

To effectively reduce the number of model parameters and enhance the real-time performance of object detection, this paper constructs the W3_D module using both Depthwise Separable Convolution (DSC) [25] and conventional convolution. Specifically, the W3_D module designed in this paper consists of the following three components:

1 × 1 Depthwise Separable Convolution: Used to reduce the number of parameters and perform channel fusion.

3 × 3 Depthwise Separable Convolution: Maintains feature extraction capabilities while significantly reducing computational costs.

Conventional Convolution Module: Further optimizes feature extraction.

**Figure 4:**
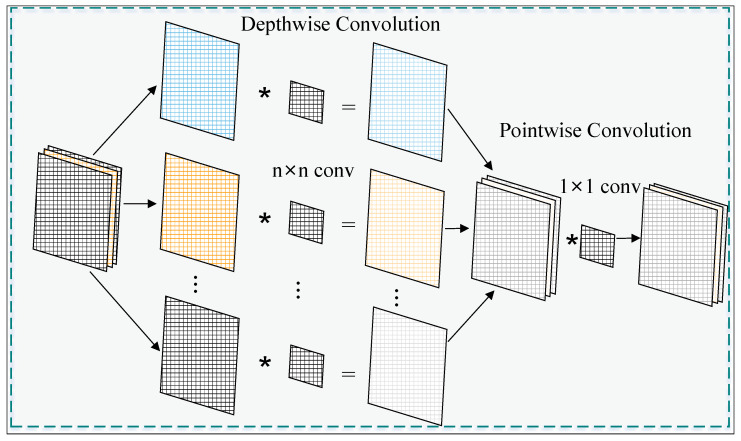
Depthwiseseparable convolution.

Depthwise Separable Convolution decomposes standard convolution into depthwise convolution and pointwise convolution. Depthwise convolution performs convolution operations independently on each channel, while pointwise convolution (1 × 1 convolution) merges the output results of depthwise convolution. Compared to standard convolution, Depthwise Separable Convolution significantly reduces computational costs and the number of model parameters while maintaining feature extraction capabilities. The process diagram of a 3 × 3 Depthwise Separable Convolution is shown in Figure 4.

### 3.2. PSCA Attention Mechanism

Traditional attention mechanisms, when measuring channel importance, mostly rely on global average or maximum pooling. However, this approach may overlook subtle differences between samples. The Position Sensitive Channel Attention (PSCA) mechanism proposed in this paper is a mechanism for enhancing the feature extraction capabilities of Convolutional Neural Networks (CNNs). By combining spatial and channel information, it performs weighting operations on feature maps, thereby improving the model’s ability to perceive important features.

The standard deviation reflects the dispersion of the data. When the standard deviation is large, the degree of dispersion of the data also increases accordingly. Traditional attention mechanisms, when measuring channel importance, mostly rely on global average or maximum pooling. However, this approach may overlook subtle differences between samples. The Position Sensitive Channel Attention (PSCA) mechanism proposed in this paper is a mechanism for enhancing the feature extraction capabilities of Convolutional Neural Networks. By combining spatial and channel information, it performs weighting operations on feature maps, thereby improving the model’s ability to perceive important features. The formula for calculating the standard deviation is:

(1) $$\mathrm{std}=\sqrt{\frac{1}{N}\sum_{i=1}^{N}{\left(x_{i}-\mu \right)}^{2}}.$$

The PSCA proposed in this paper mainly consists of two steps: position perception enhancement and channel weighting. Instead of simply calculating the average of the entire feature map, PSCA extracts the means along the height *h* and width *w* dimensions separately, thereby forming feature representations of size c×h×1 and c×1×w. The calculation formulas are as follows:

(2) $$\mu_{c}^{h}\left(h\right)=\frac{1}{w}\sum_{0\leq i\leq w}x_{c}\left(h,i\right),$$

(3) $$\mu_{c}^{w}\left(w\right)=\frac{1}{h}\sum_{0\leq j\leq h}x_{c}\left(j,w\right).$$

Here, *c*, *h*, and *w* represent the number of channels, height, and width of the feature map, respectively. At the same time, the formulas for calculating the standard deviations of the two dimensions for each channel are as follows:

(4) $$\sigma_{c}^{h}=\sqrt{\frac{1}{H}\sum_{j=1}^{H}{\left(x_{c}\left(h,j\right)-\mu_{h}\right)}^{2}},$$

(5) $$\sigma_{c}^{w}=\sqrt{\frac{1}{W}\sum_{i=1}^{W}{\left(x_{c}\left(i,w\right)-\mu_{w}\right)}^{2}}.$$

The larger the sample standard deviation, the greater the dispersion of the sample and the more pronounced its characteristics. In this paper, the mean and standard deviation of each dimension are multiplied together, which enhances the weight of the standard deviation in the feature map and strengthens the expressive power of the feature map. The purpose of the channel weighting module is to calculate the weight of each channel based on the feature map enhanced by position awareness and standard deviation. The specific steps are as follows:

1. 1D Convolutional Transformation: Use 1D convolution (Conv1d) to transform $\mu_{h}$, $\mu_{w}$, $\sigma_{h}$, and $\sigma_{w}$ to generate intermediate feature maps:
  (6) $$F_{h^{\prime }}=Conv1d\left(\mu_{h}\right),$$
  (7) $$F_{w^{\prime }}=Conv1d\left(\mu_{w}\right),$$
  (8) $$F_{\sigma h^{\prime }}=Conv1d\left(\sigma_{h}\right),$$
  (9) $$F_{\sigma w^{\prime }}=Conv1d\left(\sigma_{w}\right).$$
2. Activation Function: Apply an activation function (such as ReLU or SiLU) to the intermediate feature maps to enhance the non-linearity of the features. Here, $F_{h\prime \prime }$, $F_{w\prime \prime }\in R^{C/r\times h}$, r denotes the downsampling ratio, which is used to control the size of the module.
  (10) $$F_{h\prime \prime }=Act\left(F_{h^{\prime }}*F_{\sigma h^{\prime }}\right),$$
  (11) $$F_{w\prime \prime }=Act\left(F_{w^{\prime }}*F_{\sigma w^{\prime }}\right).$$
3. Fusion Learning: Combine the intermediate feature maps from two directions and the standard deviation feature maps to generate the final channel weight map:
  (12) $$F_{\prime }=F_{h\prime \prime }+F_{w\prime \prime }.$$
4. Channel Weighting: Use the generated channel weight map to weight the original feature map to generate the final feature map:
  (13) $$F_{out}=F\times F_{\prime }.$$

This process enables the model to adaptively learn the significance of different channels within a feature map, thus enhancing the model’s representation capabilities.

PSCA accurately assesses the importance of each channel based on the standard deviation enhanced by position perception. It can more sensitively capture the variations between samples, thereby providing clear guidance for the subsequent channel weighting module.

**Figure 5:**
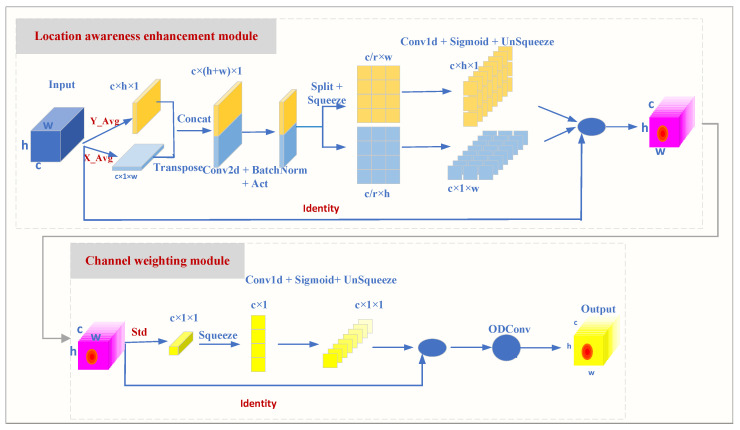
PSCA model.

The weighted feature maps are finally processed by Omni-dimensional Dynamic Convolution (ODConv) [26], which further enhances the capability of feature representation. The overall process of PSCA is shown in Figure 5.

In Figure 5, Act denotes the activation function, and the channel scaling factor r is set to 8. For the Conv2d layer, the number of input channels is *c*, the number of output channels is c/r, the kernel size is 1 × 1, the stride is 1, there is no padding, and the dilation rate is 1. Additionally, two Conv1d layers are used in this paper. These layers employ dilated convolutions to expand the receptive field and enhance position sensitivity. The parameters of these two Conv1d layers are the same: the number of input channels is c/r, the number of output channels is *c*, the kernel size is 7 × 7, the stride is 1, the padding is 6, and the dilation rate is 3. Finally, in the channel weighting module, the Conv1d layer has the same number of input and output channels, both being 1, with a kernel size of 7 × 7, a stride of 1, padding of 6, and a dilation rate of 3. In Figure 5, the overlapped pink rectangles in the upper and lower parts represent the identical feature maps after conCat fusion.

To verify the effects of different attention mechanisms and the PSCA attention mechanism on the model proposed in this paper for track fastener defect detection, comparative experiments were conducted on several attention mechanisms. The experimental results are shown in Table 2, and the bold font denotes the best values among the comparative indicators. In the subsequent tables, the bold text will represent the same meaning.

As shown in Table 2, the PSCA attention mechanism outperforms other attention mechanisms in terms of detection accuracy, number of parameters, computational load, and FPS (Frames Per Second). Therefore, the Position Sensitive Channel Attention (PSCA) mechanism proposed in this paper has a significant effect on improving the detection of track fastener defects.

### 3.3. GhostC3 Module

The network proposed in this paper introduces the lightweight GhostC3 module to replace some of the original C3 modules in the original YOLOv5 model, as indicated by the red dashed rectangular boxes in Figure 2.

**Figure 6:**
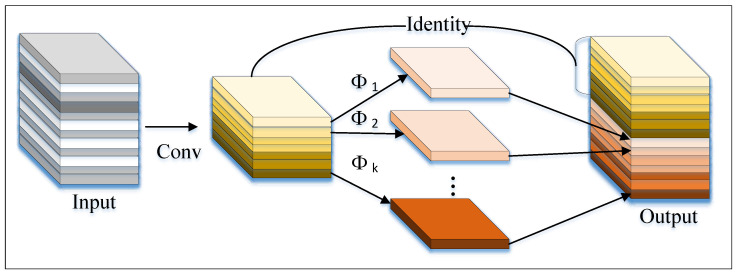
Ghost module.

GhostC3 is a lightweight Convolutional Neural Network (CNN) module designed for image classification and object detection tasks. It combines the Ghost module with the C3 module to reduce the number of parameters and computational load while maintaining model accuracy. The Ghost module splits the input feature map into two parts: the “Main Path” and the “Ghost Path”, which extract primary (column 1 after Conv) and secondary features (column 2 after Conv), respectively. Finally, the two feature maps are fused by element-wise addition. The Ghost module is illustrated in Figure 6.

### 3.4. Alpha-IoU Loss

The loss function used in YOLOv5 employs the CIoU (Complete Intersection over Union) metric, which is an advanced version of IoU that considers three geometric parameters: overlap area, center point distance, and aspect ratio. The CIoU loss function is designed to provide a more accurate measure of the difference between prediction and ground-truth bounding boxes. The formula for CIoU is as follows:

(14) $$L_{CIoU}=1-IoU+\frac{\rho^{2}\left(b,b^{gt}\right)}{c^{2}}+\beta v.$$

where:

IoU is the Intersection over Union between the prediction box and the ground-truth box.

ρ is the Euclidean distance between the center points of the prediction box and the ground-truth box.

*c* is the diagonal length of the smallest enclosing box that covers both the prediction box and the ground-truth box.

*v* is a measure of the consistency of the aspect ratios.

β is a coefficient that balances the importance of the aspect ratio term.

*b* represents the predicted bounding box.

$b^{gt}$ represents the ground-truth bounding box.

CIoU is an evaluation metric based on the Intersection over Union (IoU). When calculating the IoU between two bounding boxes, it takes into account factors such as the position, size, and shape of the bounding boxes. During the anchor box regression process, CIoU also considers three elements: the intersection ratio, the distance between the centers of the predicted and ground-truth boxes, and the aspect ratio. Different weights are assigned to these three elements. However, CIoU does not consider the very important class information between the bounding boxes.

The Alpha-IoU loss function [32] is an improvement based on IoU, and its expression is shown in Equation (15).

(15) $$L_{\alpha -IoU}=\frac{1-IoU^{\alpha }}{\alpha },\alpha >0.$$

By adjusting the parameters, it is easy to switch between different IoU_Loss functions. The current best-performing CIoU function is adopted, and a power metric α is introduced. The optimized regression loss function calculation formula is:

(16) $$L_{\alpha -CIoU}=1-IoU^{\alpha }+\frac{\rho^{2\alpha }\left(b,b^{gt}\right)}{c^{2\alpha }}+{\left(\beta v\right)}^{\alpha }.$$

Here, $\rho^{2\alpha }$ represents the IoU value between positive samples and their corresponding anchors, while $c^{2\alpha }$ represents the IoU value between negative samples and their closest anchors. Alpha-IoU introduces the parameter α, which is used to measure the class similarity and positional, size, and shape similarity between bounding boxes. By adjusting the value of α, a balance can be achieved between class and positional, size, and shape similarities, thereby better evaluating the accuracy of object detection.

This paper investigates the impact of using different Intersection over Union (IoU) metrics as localization loss functions on the accuracy of track fastener defect detection. Taking the YOLOv5s model as an example, several localization loss functions are employed and compared with the default CIoU loss of the original model. The experimental results are shown in Table 3. The experimental results show that using the Alpha-IoU loss function can more comprehensively consider the similarity between the predicted box and the ground-truth box, enabling the model to better adapt to targets of different scales during training, thereby further improving the detection accuracy of the model.

## 4. Experiments and Analysis

### 4.1. Railway Track Fastener Defect Detection Dataset and Evaluation Criteria

The original dataset used in the experiment comes from Roboflow Universe, consisting of a total of 2234 images, with 2061 images in the training set and 173 images in the test set. Anyone who wants to visit the projects or datasets of the Roboflow Universe needs to apply for a Roboflow API key. The dataset contains six types of data: fastener, fastener-2, fastener_broken, fastener2_broken, missing, and tracked_stuff. An image may contain multiple types of defects, making it difficult to accurately count the number of samples for each type of defect. Overall, the samples of the fastener-broken and fastener2-broken categories are relatively few. Due to the small and highly imbalanced nature of the dataset, several data augmentation methods were employed to improve the model’s robustness and detection accuracy. These methods include adding noise, changing brightness, cropping, translating, rotating, mirroring, and cutout. In order to increase the diversity of the samples, each image was augmented using three randomly selected methods from the aforementioned techniques. The total amount of the augmented dataset is 8936 images, with 6520 images in the training set and 2416 images in the test set.

**Figure 7:**
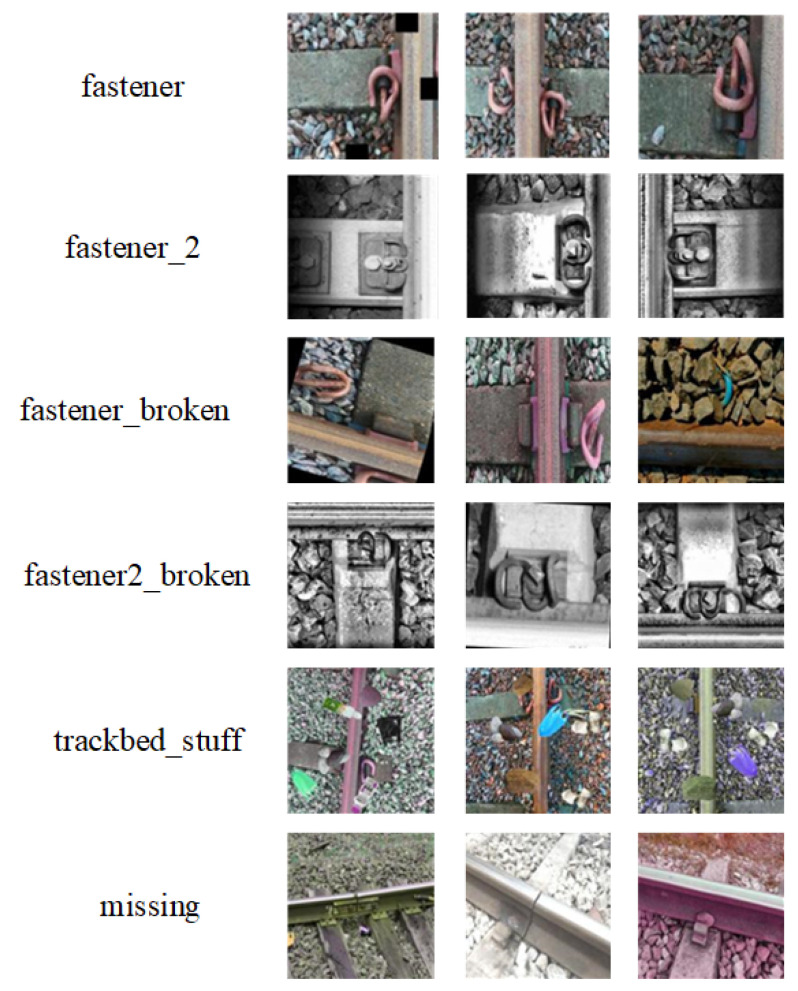
Sample Data Examples.

The original dataset includes six categories: normal fasteners (2 subclasses: fastener, fastener_2), defective fasteners (2 subclasses: fastener_broken, fastener2_broken), foreign objects (tracked_stuff), and missing fasteners (missing). Representative samples from each category are illustrated in Figure 7.

The commonly used performance evaluation indicators for railway track fastener defect detection models are detection precision, recall rate, and mean average precision (mAP). The calculation of precision is shown in Equation (17), and the calculation of recall rate is shown in Equation (18). Here, TP denotes the number of samples that are correctly predicted as positive, FP denotes the number of samples that are incorrectly predicted as positive, and FN denotes the number of samples that are incorrectly predicted as negative.

(17) $$Precision=\frac{TP}{TP+FP},$$

(18) $$Recall=\frac{TP}{TP+FN}.$$

By plotting the precision-recall curve (P-R curve) during the training process, the area under the P-R curve can be calculated. This area represents the average precision (AP) value for the corresponding target class, and the calculation method is shown in Equation (19).

(19) $$AP=\int_{0}^{1}P\left(R\right)dR.$$

Here, *R* denotes the recall rate, which is the proportion of samples that the model successfully predicts as positive among the samples that are actually positive. P(R) refers to the precision as a function of recall. mAP represents the average AP value across different classes. mAP0.5 is a measure of the mAP value of an object detection model when the IoU threshold is 0.5. mAP0.5:0.95 is a stricter evaluation metric, which calculates the average mAP value within the range of IoU thresholds from 0.5 to 0.95 (with a step size of 0.05), providing a more comprehensive assessment of the model’s performance.

### 4.2. Experimental Environment and Parameter Setting

The Linux system version used in this experiment is Ubuntu 16.04. The GPU model is RTX 3090Ti, with 24GB of video memory.The computing power resources mainly come from the AutoDL computing power leasing service platform of SightTiger Technology Co., Ltd. (Nanjing, China). The programming language is Python 3.8, and the deep learning framework is PyTorch 1.8.1. The CUDA version is CUDA 12.0. The training parameters are set as follows: the initial learning rate is 0.001, the weight decay factor is 0.0005, and the batch size is 32. The entire training process was conducted over 300 epochs using stochastic gradient descent (SGD).

### 4.3. Experimental Results and Analysis

#### 4.3.1. Ablation Experiment

In order to verify the effectiveness of each component of the improved algorithm proposed in this paper, extensive ablation experiments were conducted based on the augmented dataset. The experimental results are shown in Table 4.

As can be seen from the results, the DP-YOLO algorithm proposed in this paper achieved an mAP0.5 of 87.1% and a detection speed of 92 FPS. Compared with the baseline model, the mAP0.5 increased by 1.3%, the mAP0.5:0.95 increased by 2.8%, the model parameters decreased by 1.3%, the computational amount of the model decreased by 15.19%, and the model size decreased by 6.25%.

Among them, the main function of the C3Ghost module is to simplify the network structure to achieve the effect of lightweight. It has little effect on the improvement of mAP. Concurrently, the Alpha-IoU localization loss function mainly targets the mAP under IoU = 0.5:0.95, which can deal with fastener targets of more scales.

**Figure 8:**
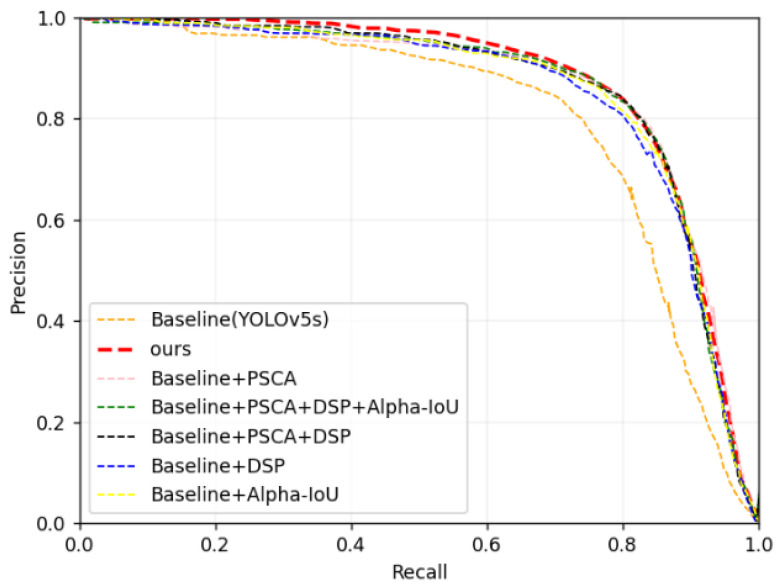
The PR curves corresponding to the ablation experiments.

Figure 8 shows the PR (precision-recall) curves of mAP0.5 for different improved models in the ablation experiments on the task of rail fastener defect detection. By comprehensively applying various improvement strategies, the final model in this paper has the largest coverage area of the horizontal and vertical axes. The PR curve coverage area of the original YOLOv5s model is the smallest. Baseline + PSCA, Baseline + DSP, and Baseline + Alpha + IoU all significantly improve the model’s recognition ability.

**Figure 9:**
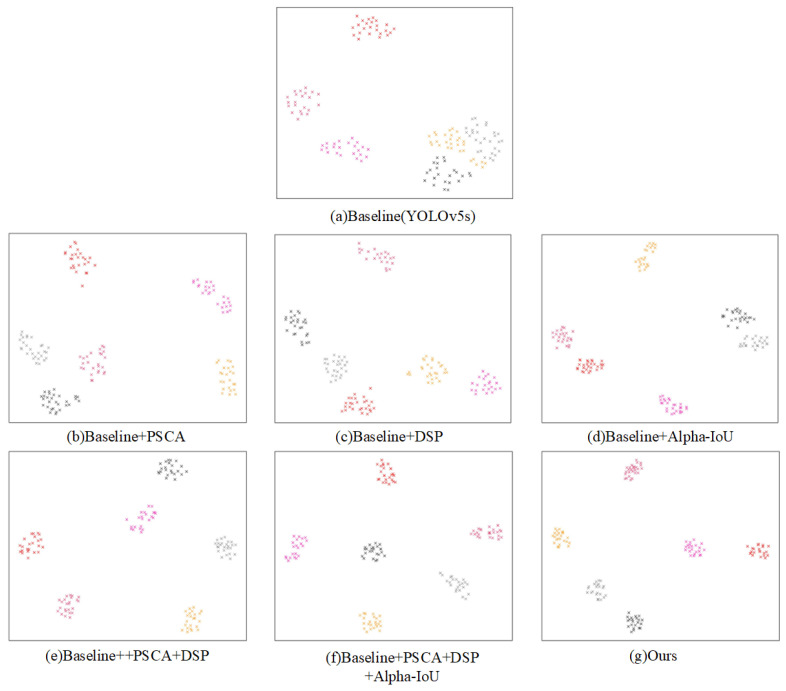
The classification capabilities of the combined model features in the ablation experiments.

Figure 9 demonstrates the classification capabilities of various combined model features in the ablation experiments.The six colors in the figure represent six different categories. As can be seen from Figure 9b,c, the sub-modules proposed in this paper have improved the classification capabilities of the Baseline model to a certain extent. Figure 9g shows that the final model proposed in this paper, DP-YOLO, extracts features with the most compact distribution within the same class of samples and the most dispersed distribution between different classes of samples.

#### 4.3.2. Comparison of Experimental Results of Different Algorithms

Table 5 presents the comparison results of the proposed algorithm in this paper with classical two-stage detection algorithms such as Faster R-CNN and Cascade R-CNN, as well as one-stage algorithms like SSD, YOLOX [33], CenterNet [34], and YOLOv7 [35]. The detection results are shown in Table 5. As can be seen from Table 5, the proposed algorithm in this paper achieves higher detection accuracy than the selected algorithms for comparison.

Table 6 presents the comparative experimental results of our improved method with other lightweight versions of YOLOv5. The results show that our model outperforms other lightweight versions of YOLOv5s in terms of the mean accuracy on the six class targets. From Table 5 and Table 6, it can be observed that the mAP0.5 value of the proposed method on the “missing” class is lower than that of the other comparison algorithms. The reason is that both the proposed DP_YOLO model and the original YOLOv5s model achieve higher detection accuracy for multi-scale and occluded targets. However, this higher detection accuracy comes at the cost of weaker generalization ability. Additionally, the dataset used in the experiment does not distinguish between the absence of the “fastener” class and the “fastener_2” class within the “missing” class data, which leads to lower detection accuracy for the “missing” class in both the DP_YOLO model and the original YOLOv5s model. Since DP_YOLO further enhances the ability to handle multi-scale targets compared to the original YOLOv5s, the detection accuracy of the DP_YOLO model on the “missing” class data is lower than that of the original YOLOv5s.

**Figure 10:**
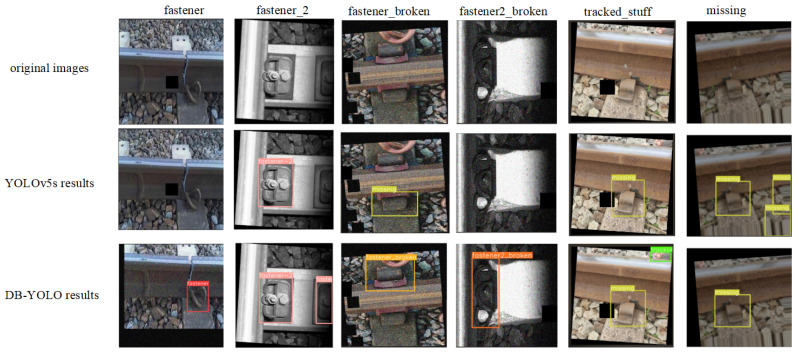
Comparison of detection results between YOLOv5s and our model.

The comparative results in Figure 10 highlight the superior detection capability of the proposed DP-YOLO algorithm over the original YOLOv5s model. Specifically, DP-YOLO not only identifies targets that YOLOv5s fails to detect but also exhibits enhanced robustness in handling complex scenarios, such as multi-scale and occluded objects (As shown in columns “fastener” and “tracked_stuff” of Figure 10). These improvements validate the effectiveness of the proposed architecture, demonstrating its potential for practical applications in track fastener defect detection.

## 5. Conclusions

To achieve real-time and accurate defect detection in resource-constrained environments, this study proposes DP-YOLO, a lightweight network based on YOLOv5s, incorporating two core innovations. First, the Depthwise Separable Convolution-enhanced (DSP) module replaces the original Bottleneck in C3 layers, reducing model parameters while enhancing multi-scale object detection capability. Second, the Position-Sensitive Channel Attention (PSCA) module adaptively weights features across spatial and channel dimensions, improving mAP0.5:0.95 by 2.1% without increasing computational overhead. Additional optimizations include integrating the C3Ghost module in the Neck (reducing FLOPs by 31%) and adopting the Alpha-IoU loss to refine bounding box regression.

Experiments on an augmented dataset (8936 images) demonstrate the effectiveness of DP-YOLO: it achieves 87.1% detection accuracy (a 1.3% improvement in mAP0.5 over the baseline YOLOv5s). These results highlight its potential for real-time deployment in industrial scenarios.

However, limitations persist, including suboptimal mAP_0.5:0.95_ (≤72.4%) due to class imbalance and complex background interference, as well as restricted generalization on rare defect categories (e.g., fastener2_broken). Semi-supervised learning (SSL) and few-shot learning (FSL) are effective methods to address this challenge, as they can utilize limited labeled data and a large amount of unlabeled data to enhance the model’s ability to recognize minority classes. Future work will prioritize semi-supervised learning to address data imbalance and multi-scale context fusion to suppress background noise, further enhancing robustness in practical applications.

## Figures and Tables

**Table 2:** The impact of different attention mechanisms on experimental results.

Attention Mechanism	mAP0.5%	Parameters/M	GFLOPs	FPS
NAM [27]	85.2	7.06	15.8	309
ECA [28]	85.9	7.06	15.8	305
CA [29]	86.0	7.04	15.8	306
CBAM [30]	86.2	8.22	15.8	298
SE [31]	86.0	7.04	15.8	310
PSCA	**86.4**	**7.04**	**15.8**	**315**

**Table 3:** The Influence of Location Loss Functions of different IoU methods on experimental results.

Algorithm	mAP0.5/%	mAP0.5:0.95/%
YOLOv5 + IoU loss	85.8	55.5
YOLOv5 + GIoU loss	85.8	55.5
YOLOv5 + DIoU loss	86.1	55.9
YOLOv5 + CIoU loss(default)	86.1	55.9
YOLOv5 + SIoU loss	**86.2**	56.1
YOLOv5 + Alpha-IOU loss	**86.2**	**56.8**

**Table 4:** Results of ablation experiments.

Improved Scheme					mAP0.5/%	mAP0.5:0.95/%	Parameters/M	GFLOPs	FPS	Model Size (MB)
Model	PSCA	DSP	C3Ghost	Alpha-IoU						
A	×	×	×	×	0.858	0.555	7.02	15.8	310	14.4
B	×	×	×	✓	0.858	0.568	7.01	15.8	310	13.7
C	×	✓	×	×	0.863	0.561	**6.89**	**13.4**	302	**13.5**
D	✓	×	×	×	0.864	0.562	7.04	15.8	**315**	13.7
E	✓	✓	×	✓	0.867	0.574	7.04	15.8	303	13.7
F	✓	✓	×	×	0.869	0.564	**6.89**	**13.4**	306	**13.5**
H	✓	✓	✓	✓	**0.871**	**0.576**	6.92	**13.4**	**315**	**13.5**

**Table 5:** Comparison of Different Target Detection Algorithms.

Model	Image-Size	mAP0.5/%						
		All	fastener	fastener-2	fastener_broken	fastener2_broken	trackbed_stuff	Missing
Faster-RCNN	640 × 640	50.2	61.5	49.6	58.4	64.6	31.5	98.2
Cascade R-CNN	640 × 640	69.3	72.3	65.4	79.6	75.5	40.4	**99.3**
SSD	512 × 512	60.1	72.2	58.4	68.4	69.9	32.4	96.8
YOLOX	640 × 640	80.3	92.4	74.3	90.5	91.2	40.3	98.4
CenterNet	640 × 640	86.9	94.7	83.6	91.1	**97.4**	42.7	98.4
YOLOv7	640 × 640	**87.1**	97.6	85.7	92.3	99.6	46.6	97.3
YOLOv5s (Baseline)	640 × 640	85.8	97.2	85.3	92.0	99.5	43.5	97.1
DP-YOLO (ours)	640 × 640	**87.1**	**97.9**	**86.7**	**92.9**	99.6	**47.3**	96.2

**Table 6:** Comparison of YOLOv5 lightweight improved models.

Model	mAP0.5/%						
	All	fastener	fastener-2	fastener_broken	fastener2_broken	trackbed_stuff	Missing
YOLOv5s (Baseline)	85.8	97.2	85.3	92.0	99.5	43.5	97.1
YOLOv5-Mobilev3s	81.1	92.4	80.2	88.4	96.2	39.6	98.6
YOLOv5-Mobilev3l	82.3	93.3	81.3	89.4	97.8	40.5	98.3
YOLOv5-ShuffleNet	81.4	92.6	80.4	88.7	96.6	40.1	98.5
YOLOv5-Ghost	85.5	96.9	83.3	92.3	99.1	42.6	98.2
YOLOv3-Tony	73.9	84.3	71.9	81.3	84.2	34.7	**99.5**
DP-YOLO (ours)	**87.1**	**97.9**	**86.7**	**92.9**	**99.6**	**47.3**	96.2

## Data Availability

No new data were created or analyzed in this study.
